# Polypropylene-Based Polymer Locking Ligation System Manufacturing by the Ultrasonic Micromolding Process

**DOI:** 10.3390/polym15143049

**Published:** 2023-07-15

**Authors:** Alex Elías-Grajeda, Elisa Vázquez-Lepe, Héctor R. Siller, Imperio Anel Perales-Martínez, Emiliano Reséndiz-Hernández, Claudia Angélica Ramírez-Herrera, Daniel Olvera-Trejo, Oscar Martínez-Romero

**Affiliations:** 1 Tecnologico de Monterrey, Institute of Advanced Materials for Sustainable Manufacturing, Av. Eugenio Garza Sada Sur 2501, Monterrey 64849, N.L., Mexico; alex.eliasgr@gmail.com (A.E.-G.); elisa.vazquez@tec.mx (E.V.-L.); emi_jose.18@hotmail.com (E.R.-H.); claudia.ramirezh@tec.mx (C.A.R.-H.); daniel.olvera.trejo@tec.mx (D.O.-T.); 2 Department of Mechanical Engineering, University of North Texas, 3940 N. Elm St., Denton, TX 76207, USA; hector.siller@unt.edu

**Keywords:** ultrasonic microinjection molding, Hem-O-Lok system, polypropylene-based polymer locking ligation system

## Abstract

In recent years, there has been a growing demand for biocompatible medical devices on the microscale. However, the manufacturing of certain microfeatures has posed a significant challenge. To address this limitation, a new process called ultrasonic injection molding or ultrasonic molding (USM) has emerged as a potential solution. In this study, we focused on the production of a specific microdevice known as Hem-O-Lok, which is designed for ligation and tissue repair during laparoscopic surgery. Utilizing USM technology, we successfully manufactured the microdevice using a nonabsorbable biopolymer that offers the necessary flexibility for easy handling and use. To ensure high-quality microdevices, we extensively investigated various processing parameters such as vibration amplitude, temperature, and injection velocity. Through careful experimentation, we determined that the microdevice achieved optimal quality when manufactured under conditions of maximum vibrational amplitude and temperatures of 50 and 60 °C. This conclusion was supported by measurements of critical microfeatures. Additionally, our materials characterization efforts revealed the presence of a carbonyl (C=O) group resulting from the thermo-oxidation of air in the plasticizing chamber. This finding contributes to the enhanced thermal stability of the microdevices within a temperature range of 429–437 °C.

## 1. Introduction

In today’s rapidly evolving landscape, the production of microinjected parts has emerged as a critical manufacturing technique with immense significance across diverse sectors, notably in the realms of medical devices. According to Whiteside et al. [1], a microdevice typically comprises a weight of just a few milligrams, with some of its features having dimensions falling within the micrometer range or its dimensional tolerances can be in the micrometer range.

There are several manufacturing technologies commonly used to manufacture microparts such as microinjection molding, micromachining, and, most recently, additive manufacturing. Among these, microinjection molding, based on transferring a thermoplastic material in the form of granules from a hopper into a heated barrel, is one of the most popular polymer processing methods due to its high productivity; however, the energy consumption is, in general, too high. Manufacturing low-volume microparts for different applications, such as customized sensors in electronics or medical devices, is a challenge in terms of dimensional accuracy. Some related problems, if tight tolerances are not achieved, are large amounts of parts being rejected, material wasted, and useless energy consumption. All these problems directly impact the part costs and affect production viability. Traditional microinjection molding involves high temperatures (and high energy consumption) (150 to 360 °C) during heating cycles which contribute to polymer degradation [2].

A novel technology called ultrasonic injection molding or just ultrasonic molding (USM) uses the energy of ultrasonic vibrations to melt and mold thermoplastic polymers. It can manufacture components with complex shapes and micrometer characteristics in short periods (seconds) [3]. The process consists of two main stages. First, a small amount of the polymer material is introduced to the plasticization chamber and energy from mechanical vibration heats and melts the material; second, the melted material is transferred to the mold cavity where it is solidified to take the specific shape.

Most of the literature review on USM technology has focused on the study and optimization of the manufacturing parameters that allow producing high-quality inject microparts on specific polymeric matrices. Michaeli et al. [4] implemented acoustic measurements, the ultrasonic reflection procedure provides information on the elastic properties of solids. In a thermoplastic compact injection molding, the ultrasonic signal provides more useful information than pressure signals (longer signals) and found a relationship between the ultrasonic signal and the shrinkage process of the molded part. Sacristán and Planellas et al. [5] did not find a relevant influence on polylactic acid (PLA) chemical degradation when clay particles are incorporated over the injection process. However, they do observe an enhancement of polybutylene succinate (PBS) degradation when an organo-modifier was employed. Grabalosa et al. [6] designed a procedure to predict the dynamics of the stepped sonotrode life by computing a map where the operating-frequencies map is related to sonotrode wear. Michaeli and Opfermann et al. [7] built a plasticizing unit, leading to a time-cycle reduction. Folgueral et al. [8] compared conventional injection molding and the USM process, finding a significant reduction in the use of raw materials from 1.686 g to 0.085 g. Vázquez et al. [9] proposed a process planning of micromilling for producing miniature mold cavities required in ultrasonic molding to reach higher precision. Sackmann et al. [2] studied the manufacture of polymer microdevices for electronic circuit boards and sensors. Young et al. [10] performed a finite-element analysis to investigate the vibration characteristics of the sonotrode and adjust the natural frequency to the desirable range to assure longitudinal vibrations only. According to the research conducted by Sato et al. [11], optical lenses can be developed through injection molding. The study found that the application of ultrasonic waves generates an oscillatory flow inside the cavity, leading to an increase in the weight of the lens. Wang et al. [12] developed a planar Bézier profiled horn cubic curve to reduce penetration force in ultrasonic cutting. Zhao et al. [13] proposed a nondestructive method for online cavity pressure measurement based on ultrasonic technology. Cheng et al. [14] developed a microultrasonic powder molding method to directly form microparts, having the advantage of including a short forming cycle, low energy consumption, and low production costs. Similarly, Luo et al. [15] used the microultrasonic powder molding process as a special semisolid forming method to fabricate semifinished microparts using 42Sn-58Bi eutectic alloy powder. The process effectively promoted the thermal performance and mechanical properties of the microparts. Liang et al. [16] developed a highly accurate hybrid process that combines ultrasonic injection molding and electrical discharge machining to manufacture microparts of GF/PP (glass fiber-reinforced polypropylene).

On the other hand, polypropylene also has been widely used to manufacture microcomponents due to the low melt-flow index. Gaxiola et al. [17] showed an efficient method to produce microspecimens using recycled polypropylene by ultrasonic microinjection molding. They performed experiments by full factorial design of experiments (DOE) combinations to reduce the morphological defects. Liang et al. [18] developed polypropylene microstructures with microgrooves surfaces, which improve hydrophobicity by microultrasonic powder molding.

Different products have been developed for medical applications, such as drug release, implants, and microfluidic devices [19]. For example, laparoscopic surgery uses a surgical stapling microdevice to ligate and repair vessels and tissues. This microdevice is named locking ligation system or Hem-O-Lok by its commercial name. The device is made with nonabsorbable biopolymers and has proper flexibility for easy use [2]. This paper focuses on finding how the process parameters of the USM, such as vibration amplitude, mold temperature, and injection velocity of the USM process affect the quality of the produced micropart. This study was complemented via chemical characterization to validate the degradation of the polymer during the USM process.

## 2. Materials and Methods

### 2.1. Materials

Polypropylene (PP-Axlene 12) with a low melt-flow index of 12 dg/min from Indelpro (Altamira, Mexico), was used for the experimental validation. The material meets the requirements of the FDA (Food and Drug Administration) accordingly to the specifications in the code of Federal Regulation title 21 CFR 117.1520. Before the material was injected, the humidity was removed by heating the pellets in an oven at 70 °C for 12 h. The Young’s modulus and yield stress of polypropylene are 561.4 MPa and 24.1 Mpa, respectively [17].

### 2.2. Component Selection

**Figure 1:**
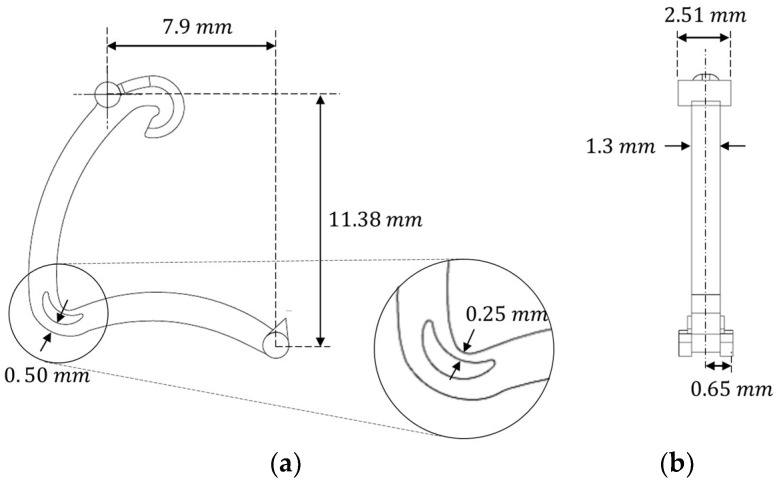
Design and nominal dimensions of the microcomponent Hem-O-Lok: (**a**) front view, (**b**) lateral view.

The locking-ligation system based on microdevice was introduced in 1999; it has medical applications in laparoscopy, such as appendectomy, cholecystectomy, and splenectomy [20]. This locking-ligation system, named Hem-O-Lok, made of a nonabsorbable polymer secures the lock engagement. This geometry has microfeatures that can efficiently be manufactured with the Ultrasonic Molding Machine. The nominal dimensions of the Hem-O-Lok are presented in Figure 1, showcasing its overall size of approximately 12 mm in length and 1.3 mm in thickness. It is worth noting that certain features within the micropart are on a much smaller scale, measured in micrometers. Figure 1 highlights the area of the part that flexes when the Hem-O-Lok is closed, indicated by the section view. There are two critical features within the microrange, which measure 0.50 mm and 0.25 mm, and, between them, there is a void that enables the staple to bend. These microcharacteristics were selected as critical because they greatly affect the proper functioning and bending of the staple.

### 2.3. Operation of Ultrasonic Molding Machine

**Figure 2:**
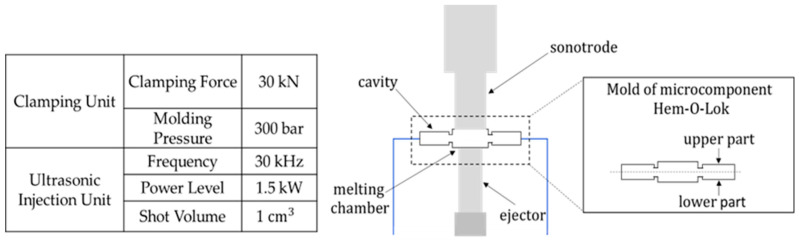
(**a**) Machine Technical Specification and (**b**) schematic diagram of the main components of the Sonorus 1G Ultrasound Molding Machine.

Sonorus 1G Ultrasound molding machine (see technical specifications in Figure 2a) was used to fabricate the Hem-O-Lok device. Figure 2b illustrates the main components of the USM machine. The sonotrode generates mechanical vibrational energy and is assembled over the melting chamber where the pellets are deposited. Around this chamber are located the mold cavities; here, the material is first injected and then solidified. The plunger, which is a cylinder placed below the melting chamber, injects the melted material into the cavities.

A production cycle consists as follows. First, the polymer pellets are manually fed into the melting chamber. Secondly, the sonotrode turns on, generating vibrational energy to heat and melt the polymer. Once the polymer is melted, the plunger injects the melted material into the mold cavities. Finally, the material solidifies, taking the shape of the mold. In the USM process, instead of having a melting temperature parameter, a certain amount of energy to generate is programmed to melt the polymeric material during a cycle.

### 2.4. Mold Design and Manufacture

**Figure 3:**
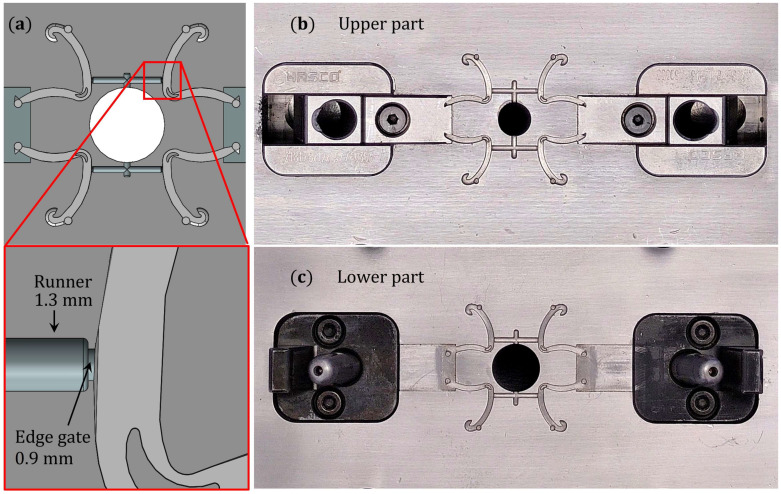
(**a**) Mold layout in which the diameter values of runner and gate are identified, optical photographs for mold (**b**) upper part and (**c**) mold lower part.

The mold (illustrated in Figure 3b,c) was manufactured using an electrical discharge machining (EDM) machine. Graphite electrodes were used for the cavities and a copper electrode for the slider features. The mold has a four-part layout and incorporates sliding units from HASCO (Z181, Z1810 & Z016) which are used to change the form of the irregular cavities. In mold design, the runners and edge gates (Figure 3a) play a critical role to guarantee the injection of the polymer into the Hem-O-Lok parts. The mold is composed of two parts, the upper and the lower part (see Figure 3b) are assembled to form the cavity of the Hem-O-Lok component and the cavity of the plasticizing chamber.

### 2.5. Ultrasonic Molding Machine Setup

Research on machine parameters for USM technology is very limited [20] and most of the previous works are limited to the effect of the main parameters (vibration amplitude and temperature). For manufacturing the Hem-O-Lok, process parameters were explored to increase part quality. First, mold plate temperatures were chosen. Then, vibration amplitude, ejector force, plunger velocity profile, plunger feeding position, and ultrasonic period were studied.

It is important to bear in mind when exploring the temperature effect that, when the temperature is very high, burrs are produced and material degrades. In addition, at higher mold temperatures, lower shrinkage is expected after cooling [21,22]. The range for each of the three factors (process parameters) used for the experiments (mold temperature, vibration amplitude, and velocity) is listed in Table 1. The temperatures for the mold were established to be 40 °C, 50 °C, and 60 °C. The vibration amplitude ranges were studied from 0.8 to 1.0 (from 80% to 100%) after a preliminary exploration where the material achieved its injection conditions. Plunger velocity was selected by screening and the velocity by increasing stages was established based on previous work with polypropylene, considering two different increasing scales [3]. In our study, we introduced three distinct plunger profiles by defining specific positions during the injection process. The corresponding positions are specified in Table 2 and each profile, namely I, II, and III, determines the linear velocity of the plunger as it reaches those designated positions.

Twenty-seven cases (case ID 1 to case ID 27) and tree replicas per case, to evaluate the repeatability, were manufactured from all combinations identified in Table 2. The pellet quantity was estimated according to the mass of the total cavity system of four simultaneous microparts, which was 447 mg. That mass is equivalent to an average of 19 pellets, as a proper dose of material.

### 2.6. Characterization Methods

The critical features of the Hem-O-Lok were measured using an InfiniteFocus Alicona Bruker microscope that uses the principle of focus variation to capture surface topography in the range from nanometers to millimeters. The geometry integrity (for checking all features were correctly injected) was validated through a stereoscope microscope.

Regarding material characterization, Fourier transform infrared (FTIR) analysis was performed with Perkin–Elmer Frontier equipment using a universal attenuated total reflectance (UATR) accessory. The infrared spectra of a Hem-O-Lok sample were measured from 4000 to 500 cm^−1^ with a resolution of 8 cm^−1^ and an average of 16 scans per sample. Thermal stability measurements were performed from room temperature to 600 °C at a heating rate of 10 °C min^−1^ with nitrogen gas flow of 20 mL/min using a Perkin–Elmer thermogravimetric analyzer equipped with Pyris 1. The mass of the microdevices was weighted in an analytical balance Ohaus Explorer of 320 g of capacity and a sensitivity of 1 mg.

## 3. Results

Since this work aims to develop the microdevices with Hem-O-Lok shape without any defects, the parameters such as plunger-velocity profile, mold temperature, and vibrational amplitude were combined to find the best processing conditions to find the best combination of values of those process parameters that produce zero-defect microparts. A total of 27 cases of process parameters were studied as a result of those combinations. The first criterion to discriminate some cases was based on the total number of completed samples obtained. Thus, from the first batch of samples made, only three cases were selected since they were the most efficient in terms of completed parts. Finally, using those selected cases (or set of parameters process), a second batch of samples was produced to determine the efficiency in terms of the number of samples obtained.

### Screening

**Figure 4:**
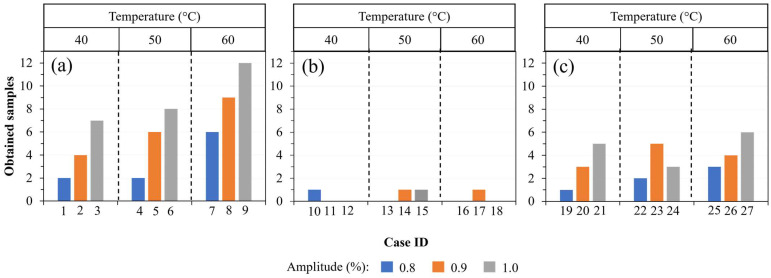
Histogram of completed samples for each case ID. Results are grouped according to different plunger velocity profiles (**a**) I, (**b**) II, and (**c**) III.

The studied condition cases were performed following the sequence listed in Table 2. For each case ID, 19 pellets were placed into the reservoir of the mold. Each case ID was manufactured three times therefore, 12 is the maximum number of samples that could be obtained on the first batch of processed experiments. Figure 4 depicts the number of obtained samples by each experiment of the first batch. As can be seen in Figure 4, in most of the experiments, less than 50% of samples were obtained. Only for case 9 were obtained the 12 samples. In experiments 6 and 8, it was possible to obtain nine and eight samples, respectively. On the contrary, no sample was obtained for cases IDs 11, 12, 13, 16, and 18. Case IDs 1–9 were made under a constant plunger velocity (I), but experiments 10–18 and 19–27 were done under a higher plunger velocity (II) and (III), respectively. It seems that with the established conditions of vibration amplitude and temperature, a constant velocity favors obtaining a higher number of samples. Case IDs 10–18, made under a plunger velocity profile II, showed the lowest efficiency (3.7%) in manufacturing the samples. Instead, the highest efficiency (51.8%) was observed for the experiment group of constant plunger velocity. In this case ID, the highest vibration amplitude (1.0) and the highest temperature (60 °C) were the best set of process parameters to obtain the maximum number of samples. It is worth mentioning that only completed polymer locking ligation samples were considered.

**Figure 5:**
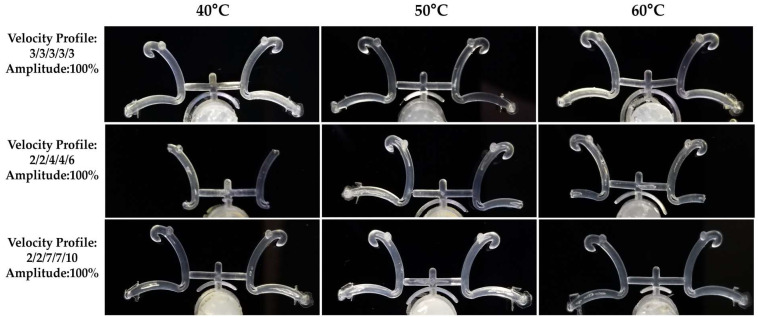
Optical pictures of polypropylene-based locking ligation samples captured by Stereoscope microscope.

Figure 5 depicts the images of locking ligation samples manufactured under experimental conditions with a vibration amplitude of 100%. Incomplete parts were produced for those experiments made under the plunger velocity profile II for the three temperatures (40, 50, and 60 °C). Some defects, such as burrs and bubbles, are observed at the edges of the locking ligation for the samples obtained at the highest velocity profile III. Fewer defects were observed for the set of experiments performed at the plunger velocity profile I.

**Figure 6:**
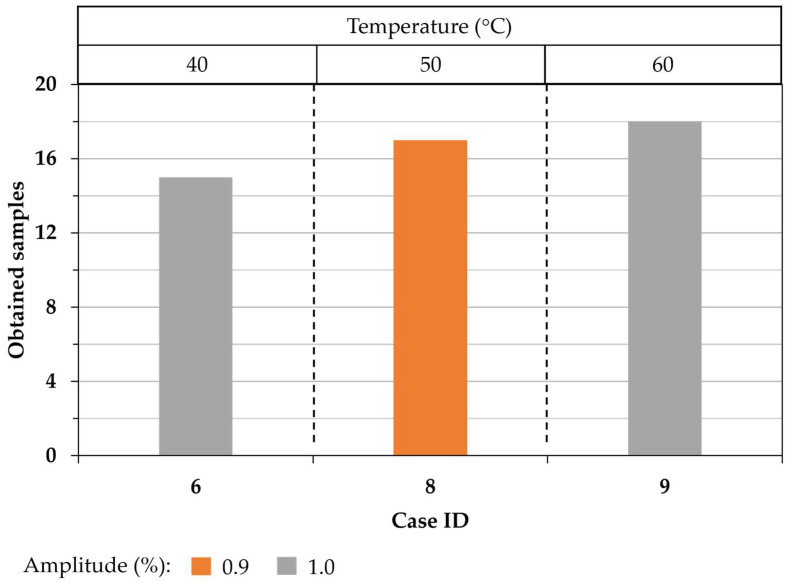
Number of completed parts for the second batch of case IDs 6, 8, and 9.

Since case IDs 6, 8, and 9 showed the highest number of completed parts, those process parameters were selected to make a second batch of samples and evaluate the repeatability of the USM technology. Five repetitions were made for each studied condition case, and the number of completed samples is shown in Figure 6. Notice a great number of samples were obtained under those conditions. In practice, a maximum of four Hem-O-Lok microparts can be obtained by each injection process; therefore, it is expected to obtain a maximum of 20 specimens by the five experiments performed. The set of process parameters of case ID 9 presented the highest number of completed parts (18 specimens), and the lowest was for the set of process parameters 6 (15 specimens). That can be interpreted as an efficiency of 90 and 75%, respectively. Notice that the maximum number of obtained samples is observed for the experiments made under a vibration amplitude of 100% ante temperature of 60°. In contrast, case ID 8 showed an efficiency of 85% for a vibration amplitude of 90%.

**Figure 7:**
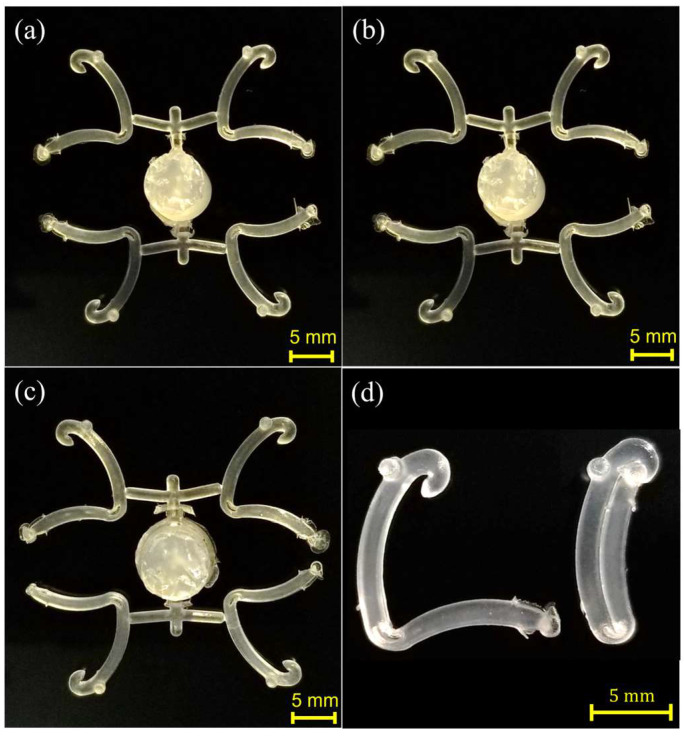
Second batch Hem-O-Lok microcomponents obtained by USM under a constant plunger velocity profile (I) and the following conditions: (**a**) 50 °C, vibration amplitude of 100%, (**b**) 60 °C, vibration amplitude of 90%, (**c**) 60 °C, vibration amplitude of 100%. (**d**) Microcomponent open and closed, showing a good adjustment and bending since no voids are observed.

In addition, of the number of microcomponents obtained for each case ID, the obtained samples show a very well-defined shape and microfeatures (defined by the mold). Figure 7 depicts four fully completed Hem-O-Lok parts obtained for case IDs 6, 8, and 9. For example, in Figure 7d, the polypropylene-based microcomponent presents no voids and, when used, good bending behavior is observed; in other words, it can be correctly closed as its function requires.

Another quality criterion of study is the mass of the injected micropart. Mass was studied because it is a parameter that can reflect the filling of the mold due to process parameters [23]. The average mass for manufactured specimens under case IDs 6, 8, and 9 was obtained from three samples. According to these results, the samples manufactured at constant plunger velocity and vibration amplitude of 100% have an average mass of 43.5 mg. In contrast, the samples obtained with a vibration amplitude of 90% and constant velocity profile have an average mass of 44.4 mg. The temperatures of 50 °C and 60 °C do not influence the mass of the Hem-O-Lok specimens since case ID 6 and 9 were manufactured under the same values of vibration amplitude and plunger velocity and they have the same average mass. It seems that the specimen mass had variations based on vibration amplitude.

**Figure 8:**
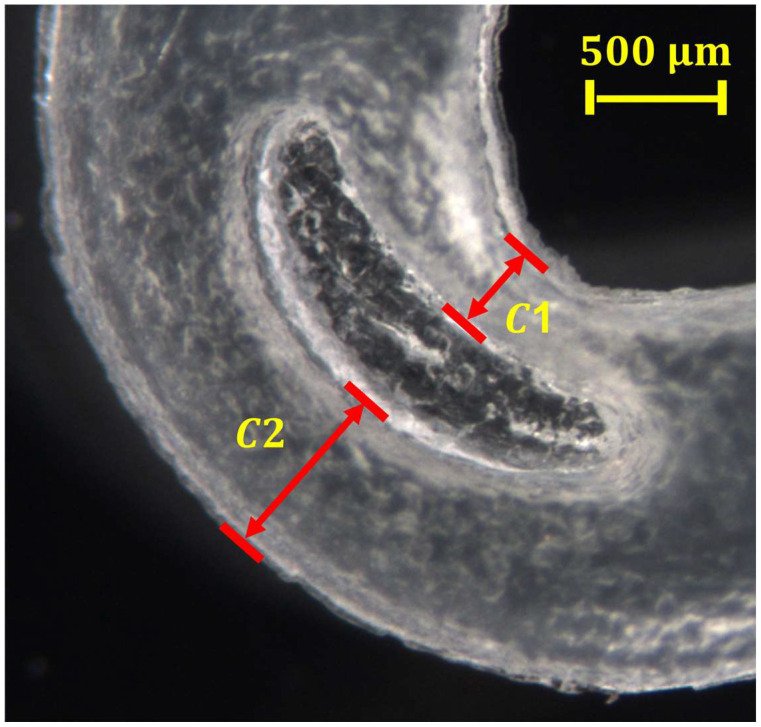
Optical microscopy image of selected critical microfeatures for comparing the nominal with the experimental dimensions of the manufactured locking ligation sample.

The volume contraction of the polymers occurs in the cooling step and lasts until the temperature of the processed piece achieved room temperature. This condition is characteristic of many polymers, such as polypropylene [23], and it is known as shrinkage. During the manufacturing process, the melting polymer is ejected from the plasticizing chamber into cavities to adopt the specific geometry. The complete filling of the cavities depends on the polymer-flow index and the screening stages as vibration amplitude, mold temperature, and plunger velocity. To evaluate shrinkage, the dimensions of two critical features were considered to monitor any defect developed. The nominal dimension was compared to the experimental dimensions, and Figure 8 illustrates the critical microfeatures, C1 and C2, of the Hem-O-Lok specimens that were selected to be compared with the mold dimensions and evaluate shrinkage. 

**Figure 9:**
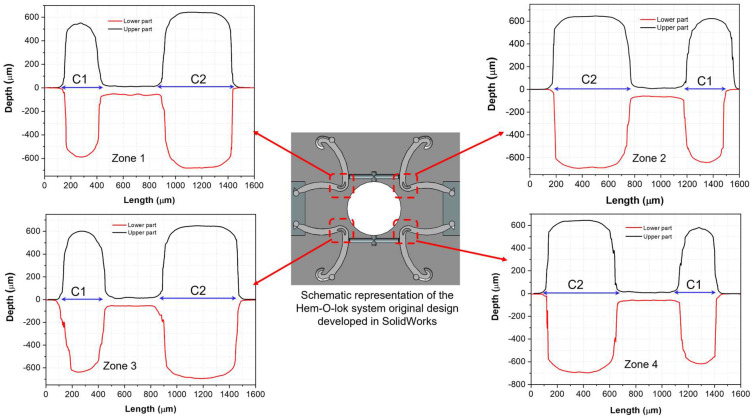
Cavities profile of the critical dimensions C1 and C2 of the Hem-O-Lok system for the four zones of mold computed by an Infinitum focus microscope.

Figure 9 shows the cavity of the mold; each area marked in red dashed lines was digitalized with the InfiniteFocus Alicona for the upper and lower mold. Table 3 shows the transversal section with the width at half high of each critical dimension for each cavity and the computed critical dimensions for locking ligation samples.

Notice that the critical microfeatures show some variations according to the mold zone in which the locking ligation sample was manufactured. For the lower part, the average dimension of C1 is 268.58 μm for the locking ligation sample and 283.85 μm for the mold, while for the upper part is 273.52 and 291.02 μm, respectively. The average critical microfeatures of C2 for the samples and mold for the lower part are 538.38 and 563.61 μm, respectively. Smaller dimensions for the manufactured samples were observed compared to the mold. This fact can be attributed to the natural shrinkage behavior of polypropylene. Nevertheless, in some zones, the dimensions of samples are not smaller than the mold, which can be due to some irregularities of the mold cavity that interfere with the highs and widths of the obtained microparts, as explained below.

According to the cavity profile for each identified mold zone (1, 2, 3, and 4), some irregularities for cavities C1 and C2 were observed and no geometric borders match between the lower and upper part; for example, C1 does not have the same dimension for parts A and B (zones 2 and 3). The most apparent irregularity is in the center between the two critical microfeatures. According to the original drawing design, this part of the mold should be closed (with no space between the upper and lower part). However, a separation of ~45.8 mm between the lower and upper part for all zones is observed, resulting in a connection between cavities C1 and C2, and producing bigger dimensions. The difference between the Hem-O-Lok specimens and mold dimensions can be attributed to this fact (see Figure 8, burrs between the C1 and C2 are observed).

**Figure 10:**
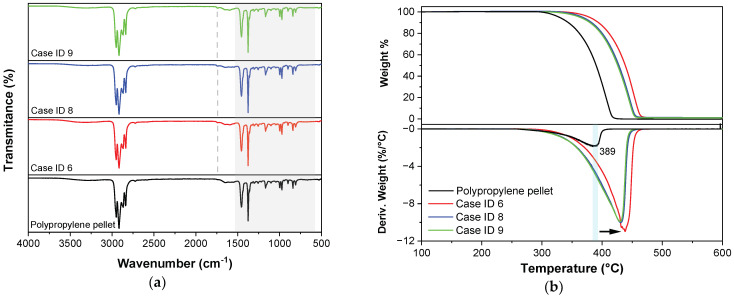
(**a**) FTIR spectra and (**b**) TGA and DTGA of Hem-O-Lok microcomponents for case IDs 6, 8, and 9, as well as the reference polymer.

Since IR radiation interacts with the molecule of a sample generating an impact over the atomic vibrations, specific absorption and/or transmission bands of energy can be obtained. Figure 10a shows FTIR spectra for the polypropylene pellet and the experimental samples computed in the mid-IR spectrum of 500 and 4500 cm^−1^. The information that can be obtained from a mid-IR spectrum is the fingerprint region of the molecular chain located from 600 to 1550 cm^−1^; the single bond region, where groups such as O–H, N–H, and C–H are presented, situated from 2500 to 4000 cm^−1^, and a double bond region from 1500 to 2000 cm^−1^. A stretching vibration corresponding to C–C is located at 808 cm^−1^ [24,25], while at 839 cm^−1^ is located the absorption peak of C–CH_3_ stretching [24]. Absorption peaks displayed at 970, 998, and 1166 cm^−1^ are assigned to –CH_3_ rocking vibration. The symmetric bending vibration mode of the –CH_3_ group is observed at 1375 cm^−1^. All the above-mentioned bands are located in the fingerprint region of the molecular chain of polypropylene and they are related to the methyl group presence in polypropylene [24,25]. The –CH_3_ asymmetric stretching vibration is identified at 2950 cm^−1^. Absorption bands at 1455, 2838, and 2917 cm^−1^ are attributed to –CH_2_– symmetric bending, –CH_2_– symmetric stretching, and –CH_2_– asymmetric stretching, respectively. A short band at 1740 cm^−1^ is attributed to a carbonyl (C=O) group due to a thermo-oxidation process that occurs when the air contained in the plasticizing chamber interacts with the polymer at high temperatures promoting oxidative degradation [26]. These results are evidence that screening stages such as vibration amplitude, mold temperature, and plunger velocity induce a modification of functional groups of the polypropylene matrix.

TGA curves for specimens developed under the case IDs 6, 8, 9, and polypropylene pellets are shown in Figure 10b. A single degradation step was observed in both the neat polymer as well the samples obtained by the USM process; however, thermal stability was increased for the experimental samples. The DTG curve for polypropylene pellets shows the maximum decomposition temperature at 389 °C (see Figure 10b), while for cases 6, 8, and 9, it was 437, 430, and 429 °C, respectively. The samples obtained with the highest vibration amplitude and 50 °C showed a higher increase in thermal stability. On the contrary, the same vibration amplitude (1.0) and the highest process temperature showed the lowest thermal stability. The effects of ultrasound on polymers involve nucleation and growth, which induces a large amount of shear force on the polymer chains causing rapid uncoiling and chain scission. The thermal stability increase of the polypropylene could be due to a rearrangement of polymeric chains arising once the Hem-O-Lok samples are solidified in the USM process and/or the bonding (C=O) created due to thermo-oxidation developed during the US manufacturing process, which requires more energy to break.

## 4. Conclusions

The Hem-O-Lok ligation systems were produced with polypropylene with a melt-flow rate of 12 dg/min. The results proved that more Hem-O-Lok specimens could be manufactured using a constant velocity profile (I). Another plunger velocity profile, (II) and (III), showed less than 50% manufacturing efficiency. The highest vibration amplitude and highest mold temperature promote the highest number of samples obtained.

Some irregularities of mold cavities were identified according to microscopy observations. Particularly, an average separation of ~45.8 μm between upper and lower molds induces the formation of burrs in the Hem-O-Lok specimens. In addition, these irregularities promote dimensional variations for the critical features. For instance, C1 and C2 features were bigger than the nominal design. Despite these defects, the repeatability of the injection process with selected parameters is demonstrated. In addition, regarding the functionality of the Hem-O-Lok specimen, when it was closed, a good adjustment for the internal edges was found since no voids were detected between them when the specimen was bending.

For the highest number of fully completed parts, process parameters were identified: vibration amplitude of 1.0 (100%), mold temperature of 60 °C, and a constant plunger velocity profile I. Additionally, a modification of the molecular chain in the polypropylene matrix after the injection was identified by a functional group of C=O, demonstrating an increase in its thermal stability.

While these findings provide valuable insights into this particular system, their direct applicability to other material compositions or microdevices may be limited. Therefore, future research should aim to explore the effects of different material compositions and other potential factors, such as mold-cavity design, to minimize irregularities and enhance part quality. Additionally, incorporating statistical analysis techniques, such as analysis of variance, will help identify significant factors and their interactions, providing valuable insights for process optimization.

## Figures and Tables

**Table 1:** Process parameters for the manufacturing of Hem-O-Lok parts.

Pellets per Shot	Plunger Force (N)	Work Pressure (bar)	Plunger Feeding Position (mm)	US Time (s)
19	6500	3	−15, −11, −8, −6, −3	4
Vibration Amplitude (%)	Mold Temperature (°C)		Plunger Velocity Profile (mm/s)	
0.8, 0.9, 1.0	40, 50, 60		I: 3, 3, 3, 3, 3	
0.8, 0.9, 1.0	40, 50, 60		II: 2, 2, 4, 4, 6	
0.8, 0.9, 1.0	40, 50, 60		III: 2, 2, 7, 7, 10	

**Table 2:** Case ID for each set of process parameters tested.

Case ID	1	2	3	4	5	6	7	8	9	10	11	12	13	14	15	16	17	18	19	20	21	22	23	24	25	26	27
Vibration Amplitude	0.8	0.9	1.0	0.8	0.9	1.0	0.8	0.9	1.0	0.8	0.9	1.0	0.8	0.9	1.0	0.8	0.9	1.0	0.8	0.9	1.0	0.8	0.9	1.0	0.8	0.9	1.0
Mold Temperature (°C)	40			50			60			40			50			60			40			50			60		
Plunger Velocity Profile (mm/s)	I									II									III								

**Table 3:** Average critical dimensions measured by InfiniteFocus Alicona for Hem-O-Lok mold and the microcomponent samples manufactured under case ID 9. All the magnitudes are in μm.

		Zone				Average	Standard Deviation
		1	2	3	4		
		Critical microfeature 1					
Microdevice samples	Lower part	268.07	268.62	268.29	269.36	268.58	0.49
	Upper part	260.00	260.02	313.85	260.20	273.52	23.28
Mold cavity	Lower part	283.93	286.22	282.71	282.55	283.85	1.47
	Upper part	237.59	343.36	313.21	269.93	291.02	40.41
		Critical microfeature 2					
Microdevice samples	Lower part	524.36	573.56	540.09	515.55	538.38	19.79
	Upper part	544.88	544.98	585.49	542.59	554.49	20.26
Mold cavity	Lower part	581.10	557.54	556.87	558.93	563.61	10.13
	Upper part	509.73	579.07	577.64	517.00	545.86	32.60

## Data Availability

Data available on request due to restrictions, e.g., privacy or ethical.

## References

[1] Whiteside B.R., Martyn M.T., Coates P.D., Allan P.S., Hornsby P.R., Greenway G. (2003). Micromoulding: Process characteristics and product properties. Plast. Rubber Compos..

[2] Sackmann J., Burlage K., Gerhardy C., Memering B., Liao S., Schomburg W.K. (2015). Review on ultrasonic fabrication of polymer micro devices. Ultrasonics.

[3] Negre P., Grabalosa J., Ferrer I., Ciurana J., Elías-Zúñiga A., Rivillas F. (2015). Study of the Ultrasonic Molding Process Parameters for Manufacturing Polypropylene Parts. Procedia Eng..

[4] Michaeli W., Starke C. (2005). Ultrasonic investigations of the thermoplastics injection moulding process. Polym. Test..

[5] Planellas M., Sacristán M., Rey L., Olmo C., Aymamí J., Casas M.T., Del Valle L.J., Franco L., Puiggalí J. (2014). Micro-molding with ultrasonic vibration energy: New method to disperse nanoclays in polymer matrices. Ultrason. Sonochem..

[6] Grabalosa J., Ferrer I., Martínez-Romero O., Elías-Zúñiga A., Plantá X., Rivillas F. (2016). Assessing a stepped sonotrode in ultrasonic molding technology. J. Mater. Process. Technol..

[7] Michaeli W., Opfermann D. (2006). Ultrasonic Plasticising for Micro Injection Moulding.

[8] Folgueral M. (2011). New Process and Machinery for Microparts Moulding Based on Ultrasound Excitation. Final Rep. Sonoplast.

[9] Vázquez E., Amaro A., Ciurana J., Rodríguez C.A. (2015). Process planning considerations for micromilling of mould cavities used in ultrasonic moulding technology. Precis. Eng..

[10] Seo Y.S., Park K. (2012). Direct patterning of micro-features on a polymer substrate using ultrasonic vibration. Microsyst. Technol..

[11] Sato A., Ito H., Koyama K. (2009). Study of application of ultrasonic wave to injection molding. Polym. Eng. Sci..

[12] Wang D.A., Nguyen H.D. (2014). A planar Bézier profiled horn for reducing penetration force in ultrasonic cutting. Ultrasonics.

[13] Zhao P., Wang S., Ying J., Fu J. (2013). Non-destructive measurement of cavity pressure during injection molding process based on ultrasonic technology and Gaussian process. Polym. Test..

[14] Wu S.Y., Wu X.Y., Xu B., Cheng R., Luo F., Ruan S.C. (2014). A micro-ultrasonic powder moulding method to fabricate Sn-Bi alloy micro parts. J. Mater. Process. Technol..

[15] Luo W.Y., Wu X.Y., Wu S.Y., Xu B., Cheng R., Ruan S.C. (2015). Micro-ultrasonic powder moulding of Sn-Bi/Cu composite micro parts in semisolid form. J. Mater. Process. Technol..

[16] Liang X., Liu Y., Liu Z., Ma J., Zhang Z., Ruan W., Ren S., Peng T., Wu X., Shi H. (2021). Materials & Design Ultrasonic injection molding of glass fiber reinforced polypropylene parts using tungsten carbide-cobalt mold core. Mater. Des..

[17] Gaxiola-Cockburn R., Martínez-Romero O., Elías-Zúñiga A., Olvera-Trejo D., Reséndiz-Hernández J.E., Soria-Hernández C.G. (2020). Investigation of the mechanical properties of parts fabricated with ultrasonic micro injection moldingprocess using polypropylene recycled material. Polymers.

[18] Liang X., Liu Y., Ma J., Gong F., Lou Y., Fu L., Xu B. (2020). Fabrication of micro ultrasonic powder molding polypropylene part with hydrophobic patterned surface. Materials.

[19] Heredia-Rivera U., Ferrer I., Vázquez E. (2019). Ultrasonic molding technology: Recent advances and potential applications in the medical industry. Polymers.

[20] Grabalosa J., Ferrer I., Elías-Zúñiga A., Ciurana J. (2016). Influence of processing conditions on manufacturing polyamide parts by ultrasonic molding. Mater. Des..

[21] Estrada P., Siller H.R., Vázquez E., Rodríguez C.A., Martínez-Romero O., Corona R. (2016). Micro-injection Moulding of Polymer Locking Ligation Systems. Procedia CIRP.

[22] Rännar L.-E. (2008). On Optimization of Injection Molding Cooling. Ph.D. Thesis.

[23] Ko´sciuszko A.K., Marciniak D., Sykutera D. (2021). Post-Processing Time Dependence of Shrinkage and Mechanical Properties of Injection-Molded Polypropylene. Materials.

[24] Gopanna A., Mandapati R.N., Thomas S.P., Rajan K., Chavali M. (2019). Fourier transform infrared spectroscopy (FTIR), Raman spectroscopy and wide-angle X-ray scattering (WAXS) of polypropylene (PP)/cyclic olefin copolymer (COC) blends for qualitative and quantitative analysis. Polym. Bull..

[25] Fang J., Zhang L., Sutton D., Wang X., Lin T. (2012). Needleless melt-electrospinning of polypropylene nanofibres. J. Nanomater..

[26] Sánchez-Sánchez X., Hernández-Avila M., Elizalde L.E., Martínez O., Ferrer I., Elías-Zuñiga A. (2017). Micro injection molding processing of UHMWPE using ultrasonic vibration energy. Mater. Des..

